# Immune checkpoint inhibitors combined with targeted therapy for long-term survival in advanced pulmonary squamous cell carcinoma after first-line failure: A case report and literature review

**DOI:** 10.1097/MD.0000000000042724

**Published:** 2025-06-20

**Authors:** Caiping Ke, Manjie Li, Chunning Zhang, Qiwen Huang, Yongquan Deng, Junfen Cheng

**Affiliations:** a The First School of Clinical Medicine, Guangdong Medical University, Zhanjiang, Guangdong Province, China; b Department of Oncology, Unit 1, Maoming People’s Hospital, Maoming, Guangdong Province, China; c Department of Medical Oncology, Unit 1, Central Hospital of Guangdong Provincial Nongken, Zhanjiang, Guangdong Province, China; d Department of Pathology, Maoming People’s Hospital, Maoming, Guangdong Province, China; e Department of Pulmonary and Critical Care Medicine, The Second Affiliated Hospital of Guangdong Medical University, Zhanjiang, Guangdong Province, China.

**Keywords:** anlotinib, camrelizumab, immune checkpoint inhibitors, non-small-cell lung cancer (NSCLC), squamous cell carcinoma, targeted therapy

## Abstract

**Rationale:**

Advanced squamous cell carcinoma (SCC) of the lung remains a major clinical challenge due to limited therapeutic options, particularly in the post-immunotherapy setting. Combining immune checkpoint inhibitors with small-molecule multi-targeted tyrosine kinase inhibitors that include anti-angiogenic effects offers a promising approach to overcome treatment resistance and improve survival outcomes.

**Patient concerns:**

A 44-year-old female with a 2-year history of recurrent cough and sputum production presented with worsening symptoms over the past month, including increased cough frequency and sputum volume. No significant systemic symptoms, such as hemoptysis or dyspnea, were reported.

**Diagnoses:**

Chest computed tomography revealed a 35 mm × 31 mm mass in the right middle lung with enlarged supraclavicular and mediastinal lymph nodes. A biopsy confirmed the diagnosis of SCC. Staging was determined as cT3N3M1a (stage IVA), and genetic testing revealed no actionable driver mutations, while PD-L1 expression was 30% (tumor proportion score).

**Interventions:**

The patient initially received first-line treatment with pembrolizumab combined with docetaxel and nedaplatin, achieving partial response. After progression, second-line therapy included gemcitabine and cisplatin chemotherapy with synchronous radiotherapy, followed by camrelizumab and anlotinib. Regular imaging follow-ups guided therapy adjustments, including extended dosing intervals for camrelizumab during disease stabilization.

**Outcomes:**

Over 4 years of treatment, the patient achieved durable partial response, with significant reduction in tumor burden and no new metastases. As of the most recent follow-up, the patient exhibited an overall survival of 59 months and progression-free survival of 51 months for second-line therapy, with manageable adverse effects including secondary hypothyroidism and grade 2 hypertension.

**Lessons:**

This case underscores the potential efficacy of combining immune checkpoint inhibitors with small-molecule multi-targeted tyrosine kinase inhibitors in treating advanced SCC of the lung after progression on first-line therapy. The complementary mechanisms of immune modulation and tumor microenvironment normalization may offer an effective strategy for addressing immune resistance in SCC.

## 1. Introduction

Lung cancer is one of the most prevalent malignancies globally and the leading cause of cancer-related mortality. According to the World Health Organization, approximately 1.8 million deaths from lung cancer occurred worldwide in 2020, accounting for 18.7% of all cancer-related deaths.^[1]^ In China, lung cancer also remains the primary cause of cancer-related death, with an estimated 710,000 fatalities in 2022, constituting 23.8% of all cancer deaths in the country.^[2]^ Non-small-cell lung cancer (NSCLC) accounts for approximately 85% of all lung cancer cases, with squamous cell carcinoma (SCC) being one of the most common histological subtypes. Globally, over 400,000 deaths annually are attributed to lung SCC.^[3]^ By the time of diagnosis, most patients present with advanced-stage disease, making surgical interventions unfeasible.

Recent advancements in the treatment of advanced squamous cell lung cancer have been driven by the use of immune checkpoint inhibitors (ICIs) in combination with chemotherapy. The publication of the KEYNOTE-407 study marked a significant breakthrough, addressing the longstanding lack of progress in the management of advanced squamous lung cancer.^[4]^ Based on the results of this pivotal study, the National Comprehensive Cancer Network (NCCN) Clinical Practice Guidelines have recommended pembrolizumab in combination with platinum-based chemotherapy as the preferred first-line treatment for patients with advanced squamous NSCLC and an Eastern Cooperative Oncology Group (ECOG) performance status (PS) of 0 to 1.^[5]^ In November 2019, the subgroup analysis from the Chinese cohort of the KEYNOTE-407 trial, presented at the ESMO Asia Congress, further validated the global findings, demonstrating that pembrolizumab combined with chemotherapy significantly improved both overall survival (OS) and progression-free survival (PFS) in patients with advanced squamous NSCLC.^[6,7]^

Despite these advancements in first-line therapy, treatment options remain limited following disease progression in NSCLC, and overall efficacy still has considerable room for improvement. Indeed, the therapeutic landscape for second-line treatment presents significant challenges. Conventional chemotherapy, such as docetaxel, while a common option post-first-line failure, provides only modest median PFS (mPFS), reported between approximately 4.1 to 5.5 months,^[8]^ and its utility is often constrained by considerable side effects that adversely impact patient quality of life.^[9]^ Although the advent of immunotherapy, exemplified by agents like nivolumab demonstrating an OS advantage compared to docetaxel,^[10]^ marked a step forward, clinical benefits remain inconsistent. Efficacy can be influenced by factors such as PD-L1 expression status, and a substantial proportion of patients exhibit only modest responses or derive no clinical benefit.^[11]^ Underscoring this unmet need, efficacy data reveal a median objective response rate (ORR) of merely ~6.8% with second-line treatments, highlighting the urgent requirement for enhanced therapeutic strategies.^[12]^ Compounding these challenges is the growing understanding that resistance to current therapies, including ICIs, is deeply rooted in the complex biology of the tumor and its microenvironment (TME). The TME, comprising diverse cellular and noncellular components, actively contributes to cancer progression, metastasis, and therapeutic resistance.^[13]^ Key drivers of resistance include metabolic reprogramming, such as the heightened glycolysis characteristic of the Warburg effect, which fuels tumor growth, invasion, and metastasis,^[14]^ and the pervasive intratumoral hypoxia, tightly regulated by factors like HIF-1, which orchestrates adaptive responses including angiogenesis and further metabolic shifts.^[15]^ Furthermore, the immune landscape itself is highly complex; the efficacy of ICIs can be modulated by the pleiotropic effects of cytokines like TNFα within the TME^[16]^ and is influenced by the heterogeneity and functional plasticity of cytotoxic T lymphocytes (CTLs), including diverse subsets beyond conventional CD8 + cells, whose activation status and cytotoxic potential vary significantly.^[17]^ Even nonconventional immune cells like γδ T cells, while possessing antitumor potential, can exhibit exhaustion within the TME, potentially limiting ICI responsiveness unless specifically addressed.^[18]^ Processes such as the Epithelial-Mesenchymal Transition, often induced by TME signaling, further enhance tumor cell motility and invasiveness, contributing significantly to metastasis and resistance.^[13]^ This intricate biological landscape, characterized by metabolic alterations, TME heterogeneity, complex immune cell dynamics, and adaptive resistance pathways, collectively imposes a significant therapeutic ceiling on existing second-line approaches. While current clinical guidelines increasingly support combination therapies involving chemotherapy and immunotherapies (e.g., pembrolizumab), robust long-term clinical data substantiating their effectiveness, particularly in patient populations with low PD-L1 expression, remain sparse.^[19]^ Additionally, squamous cell lung cancer lacks the presence of actionable driver mutations, which restricts the use of targeted therapies. Furthermore, patient-specific factors, including advanced age and baseline PS – which can heighten toxicity concerns with conventional chemotherapeutic agents^[20]^ – alongside observed real-world treatment heterogeneity where therapeutic choices may diverge from clinical guidelines due to varied access to novel agents or participation in clinical trials,^[21]^ further complicate the management landscape. As a result, the survival prognosis for most patients remains poor. Existing reports suggest that the median OS for patients with advanced squamous NSCLC typically ranges from 8 to 14 months, with the median PFS generally being <6 months.^[22–24]^

This report presents a case of an advanced squamous cell lung cancer patient who achieved over 4 years of survival after progression on first-line treatment, following second-line therapy with the combination of camrelizumab and anlotinib. The purpose of this case report is to enhance understanding of the efficacy of ICIs combined with small-molecule multi-targeted tyrosine kinase inhibitors in the real-world treatment of NSCLC after first-line failure. Through the analysis of this case, we aim to provide new insights into second-line therapy for advanced squamous cell lung cancer and contribute valuable clinical evidence to this field.

## 2. Case presentation

### 2.1. Patient information

A 44-year-old female presented on January 14, 2020, with a 2-year history of recurrent cough and sputum production. Initially, her symptoms were intermittent, accompanied by small amounts of white frothy sputum. Over the past month, however, her cough frequency increased, and she experienced a notable increase in sputum volume. The patient denied hemoptysis, chest pain, or dyspnea. She had no significant medical history, no smoking history, no family history of cancer, and no known exposure to harmful chemicals.

### 2.2. Clinical presentation and auxiliary examinations

Upon admission, the patient’s ECOG PS score was 0, indicating that her general physical condition was good. Physical examination revealed an enlarged lymph node in the right supraclavicular region, approximately 2 cm × 2 cm in size, with a firm consistency, poor mobility, and a smooth surface. No other enlarged superficial lymph nodes were palpated. On lung examination, breath sounds were clear in both lungs, with no dry or wet crackles detected. Cardiovascular and abdominal examinations were unremarkable. Laboratory tests showed no abnormalities in the complete blood count, with a mildly elevated C-reactive protein (CRP), while liver and renal functions were normal.

**Figure 1.**
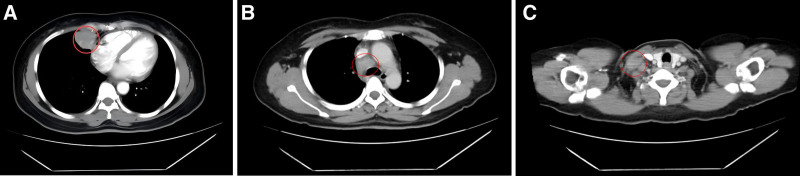
Baseline chest CT. (A) Lesion in the right lung; (B) enlarged mediastinal lymph nodes; (C) metastatic lymph node in the right supraclavicular region. CT = computed tomography.

A chest computed tomography (CT) scan performed on January 14, 2020, revealed a soft tissue mass in the right middle lung, approximately 35 mm × 31 mm in size, with ill-defined borders and uniform density (Fig. 1A). The CT values ranged from 44 to 51 HU. The lesion showed heterogeneous enhancement, with CT values of 72 to 96 HU. Additionally, a small cavity, approximately 8 mm × 5 mm in size, was observed in the right middle lung (Fig. 1B), with a well-defined border and enhancement at the lesion’s edge. Multiple enlarged lymph nodes were identified in the bilateral supraclavicular and mediastinal regions, some of which were fused into masses, showing strong enhancement on contrast imaging (Fig. 1C). No pleural effusion was observed, and no bony chest wall involvement was evident.

**Figure 2.**
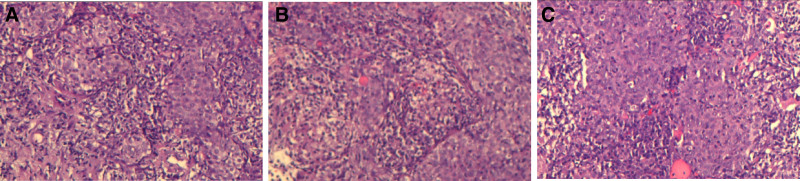
Percutaneous lung biopsy pathology show (×200). Stained section of SCC, showing irregular, infiltrative clusters of atypical squamous epithelial cells with pronounced nuclear pleomorphism, as well as evidence of keratin pearl formation and intercellular bridges. SCC = squamous cell carcinoma.

On January 23, 2020, a biopsy of the right middle lung mass was performed, and pathology confirmed the diagnosis of SCC (Fig. 2A–C). Immunohistochemical staining results were as follows: Vimentin (−), CK (+), CK7 (−), TF1 (−), CD56 (−), CK5/6 (+), Syn (−), Ki-67 (30%+), P63 (+). Genetic testing did not reveal common driver mutations, including EGFR, ALK, and ROS1. PD-L1 expression was 30% (tumor proportion score [TPS]) and 50% (combined positive score) using the 22C3 antibody.

Genetic testing revealed that the patient was negative for common driver mutations, including EGFR, ALK, and ROS1 (Table 1). The PD-L1 expression was found to be 30% (TPS) (Table 2). A whole-body bone scintigraphy (ECT) showed no abnormalities, and magnetic resonance imaging (MRI) of the brain did not reveal any significant parenchymal lesions. Laboratory tests, including the complete blood count, liver and renal function, and tumor markers, were all within normal limits. Furthermore, the hepatitis B surface antigen was negative (Fig. S1, Supplemental Digital Content, https://links.lww.com/MD/P111).

**Table 1:** Genetic sequencing test results.

Gene	Test content	Test results (frequency/copy number)
EGFR	Exon 18	**–**
	Exon 19	**–**
	Exon 20 (including T790M)	**–**
	Exon 21	**–**
ERBB2 (HER2)	Amplification	**–**
	Mutation	**–**
ALK	Rearrangement	**–**
ROS1	Rearrangement	**–**
MET	Amplification	**–**
	Exon 14 skipping	**–**
RET	Rearrangement	**–**
BRAF	Codon 600	**–**
KIT	Exon 9	**–**
	Exon 11	**–**
	Exon 13	**–**
	Exon 17	**–**
PDGFRA	Exon 12	**–**
	Exon 18	**–**
BRCA1	Mutation	**–**
BRCA2	Mutation	**–**
KRAS	Codon12/13/59/61/117/146	**–**
	Mutations at other loci	**–**
NRAS	Codon12/13/59/61/117/146	**–**
	Mutations at other loci	**–**
NTRK1	Rearrangement	**–**
PIK3CA	Mutation	**–**

The “–” indicates that no mutations related to targeted therapy were found in this test.

**Table 2:** PD-L1 IHC testing report.

Test item	Antibody type	Test method	Test result
PD-L1 IHC	22C3	IHC	PD-L1 TPS 30% CPS 50%

CPS = combined positive score, IHC = immunohistochemistry, PD-L1 = programmed death-ligand 1.

### 2.3. Diagnostic assessment

The final diagnosis was right middle lung SCC, staged as cT3N3M1a (stage IVA). The patient had no smoking history, no family history of cancer, and no prior history of tuberculosis or related diseases.

### 2.4. Treatment course and therapeutic outcome assessment

#### 2.4.1. *February 2, 2020*

The patient was administered a first-line treatment regimen consisting of docetaxel (75 mg/m^2^; *Jiangsu Aosaikang Pharmaceutical Co., Ltd., National Drug Approval Number: H20064301*), nedaplatin (80 mg/m^2^; Qilu Pharmaceutical Co., Ltd., National Drug Approval Number: H20050562), and pembrolizumab (200 mg; *Merck & Co., Registration Certificate Number: S20180019*) administered every 3 weeks, for a total of 2 cycles.

#### 2.4.2. *March 17, 2020*

**Figure 3.**
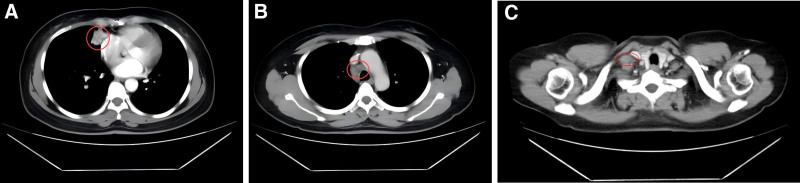
Chest CT follow-up on March 17, 2020. (A) Significant reduction in the right lung lesion; (B) reduction in the size of the enlarged mediastinal lymph nodes; (C) reduction in the size of the metastatic lymph node in the right supraclavicular region. CT = computed tomography.

A follow-up chest CT scan revealed that the primary tumor in the right middle lung (approximately 29 mm × 21 mm) and most of the enlarged lymph nodes had reduced in size (Fig. 3A–C), demonstrating an initial response to treatment. No significant adverse reactions were observed during this period. The patient continued with the combination therapy of docetaxel, nedaplatin, and pembrolizumab.

#### 2.4.3. June 11, 2020

**Figure 4.**
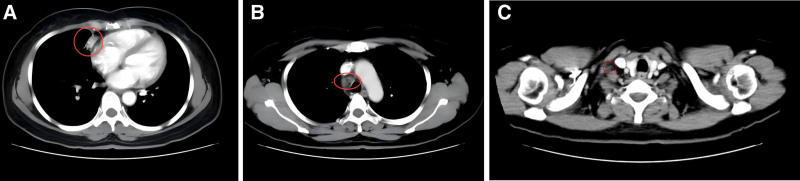
Chest CT follow-up on June 11, 2020. (A) Significant reduction in the right lung lesion; (B) further reduction in the size of the enlarged mediastinal lymph nodes; (C) further reduction in the size of the metastatic lymph node in the right supraclavicular region. CT = computed tomography.

After 6 cycles of treatment, a follow-up chest CT scan showed that the primary tumor in the right middle lung (approximately 24 mm × 15 mm) and some of the lymph node metastases continued to decrease in size (right supraclavicular lymph node approximately 11 mm × 8 mm) (Fig. 4A–C). Pembrolizumab (200 mg) was initiated as maintenance monotherapy, administered every 3 weeks for a total of 3 cycles.

#### 2.4.4. September 9, 2020

**Figure 5.**
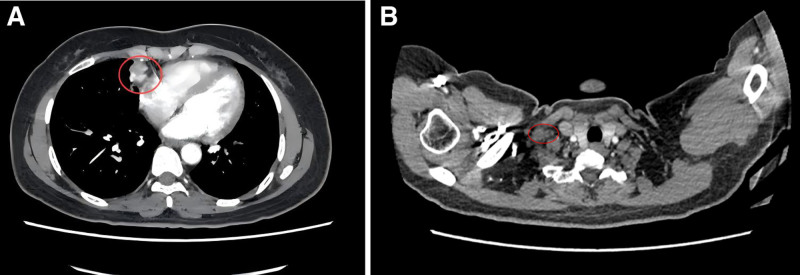
Chest CT follow-up on September 9, 2020. (A) Slight enlargement of the right lung lesion compared to June 11; (B) enlargement of the right supraclavicular lymph node. CT = computed tomography.

A follow-up chest CT showed the primary tumor in the right middle lung (approximately 32 mm × 18 mm) and enlarged lymph nodes (right supraclavicular lymph node approximately 15 mm × 12 mm) (Fig. 5A and B), indicating disease progression (PD). The PFS for first-line treatment was 7.5 months. No metastases were observed in whole-body bone scintigraphy, brain MRI, or abdominal CT. Systemic chemotherapy with sequential pulmonary lesion and synchronous radiotherapy was administered. The regimen consisted of gemcitabine (*Jiangsu Hansoh Pharmaceutical Group Co., Ltd., National Medicine License No. H20030104*) 1000 mg/m^2^ on days 1 and 8 (d1, d8), and cisplatin (Yunnan Plant Pharmaceutical Co., Ltd., National Medicine License No. H53021741) 30 mg/m^2^ on days 1 to 3 (d1–d3) for 2 cycles. The IMRT radiotherapy dosage was: GTV: 64 Gy/32 f, GTVnd: 64 Gy/32 f, and CTV: 54 Gy/32 f.

#### 2.4.5. October 30, 2020

**Figure 6.**
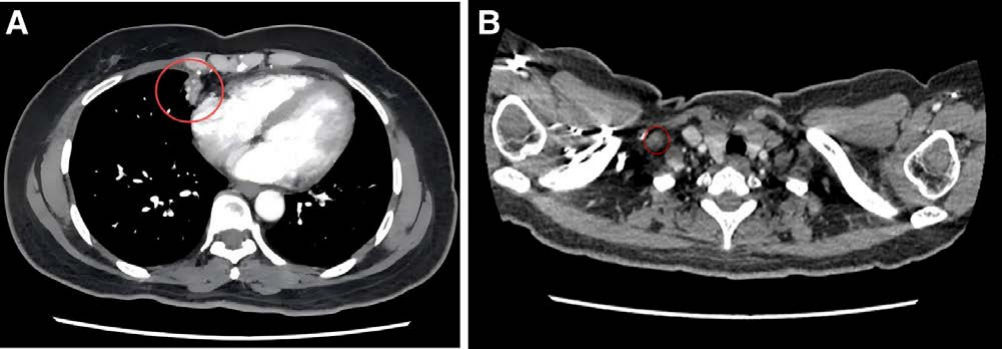
Chest CT follow-up on October 30, 2020. (A) Significant reduction in the right lung lesion compared to the previous scan; (B) significant reduction in the size of the right supraclavicular lymph node. CT = computed tomography.

A follow-up chest CT showed that the primary tumor in the right middle lung (approximately 28 mm × 12 mm) and some lymph node metastases had decreased in size (right supraclavicular lymph node approximately 10 mm × 10 mm) (Fig. 6A and B).

#### 2.4.6. January 20, 2021

**Figure 7.**
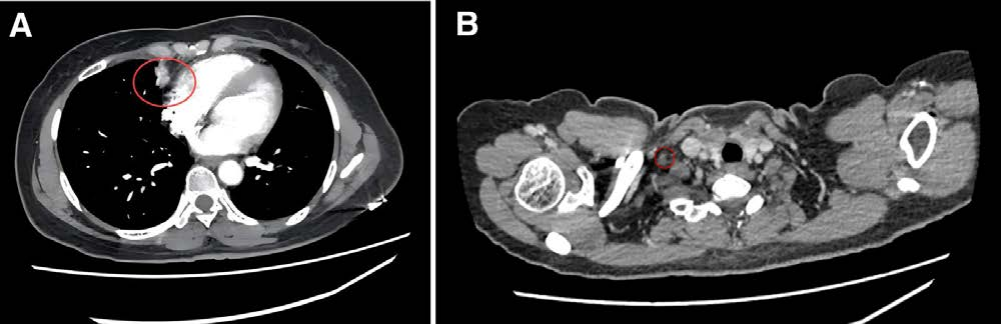
Chest CT follow-up on January 20, 2021. (A) Continued reduction in the right lung lesion; (B) continued reduction in the right supraclavicular lymph node. CT = computed tomography.

One month after completing radiotherapy, a follow-up chest CT showed that the right middle lung tumor (approximately 21 mm × 11 mm) (Fig. 7A) and enlarged lymph nodes had continued to decrease in size (right supraclavicular lymph node approximately 8 mm × 8 mm) (Fig. 7B). The treatment regimen was switched to camrelizumab (*Jiangsu Hengrui Medicine Co., Ltd., National Medicine License No. S20190027*) 0.2 g every 3 weeks in combination with anlotinib (*Zhejiang Chia Tai Tianqing Pharmaceutical Co., Ltd., National Medicine License No. H20180004*) 12 mg daily from day 1 to day 14 (d1–d14), administered for 4 cycles.

#### 2.4.7. March 24, 2021

**Figure 8.**
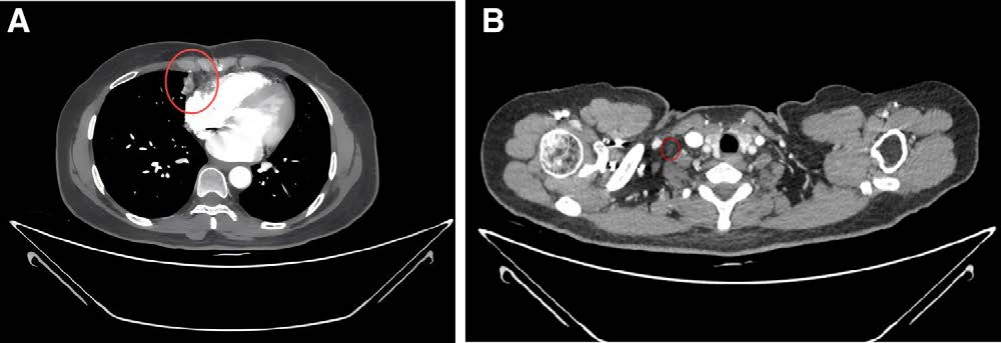
Chest CT follow-up on March 24, 2021. (A) Further reduction in the right lung lesion; (B) further reduction in the right supraclavicular lymph node.

A follow-up chest CT showed that the primary tumor in the right middle lung (approximately 17 mm × 10 mm) (Fig. 8A) and the lymph nodes (right supraclavicular lymph node approximately 7 mm × 7 mm) (Fig. 8B) continued to decrease in size. The combination of camrelizumab 0.2 g every 3 weeks and anlotinib 12 mg daily continued, with stable treatment effects. No new metastases were observed during regular follow-ups.

#### 2.4.8. July 8, 2022

**Figure 9.**
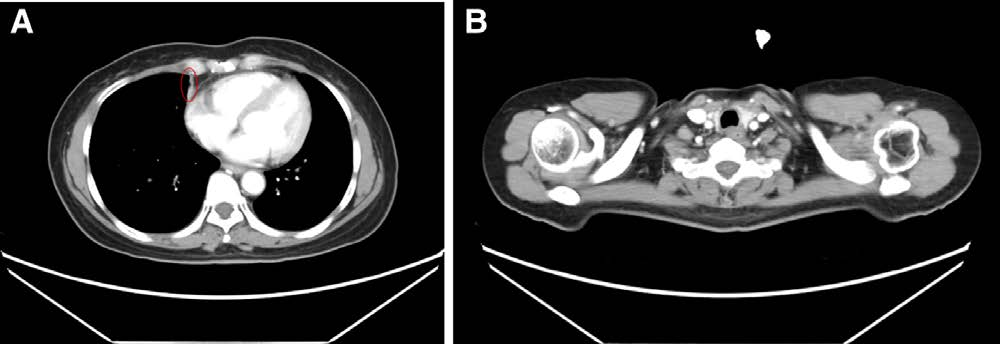
Chest CT follow-up on July 8, 2022. (A) Continued reduction in the right lung lesion; (B) no detectable metastatic lesion in the right supraclavicular lymph node. CT = computed tomography.

A follow-up chest CT showed that the primary tumor in the right middle lung (approximately 17 mm × 6 mm) had slightly reduced in size (Fig. 9A), while there were no signs of supraclavicular lymph node metastasis (Fig. 9B). The combination of camrelizumab 0.2 g every 3 weeks and anlotinib 12 mg daily continued. No new metastatic lesions were observed during regular follow-ups.

#### 2.4.9. March 23, 2023

**Figure 10.**
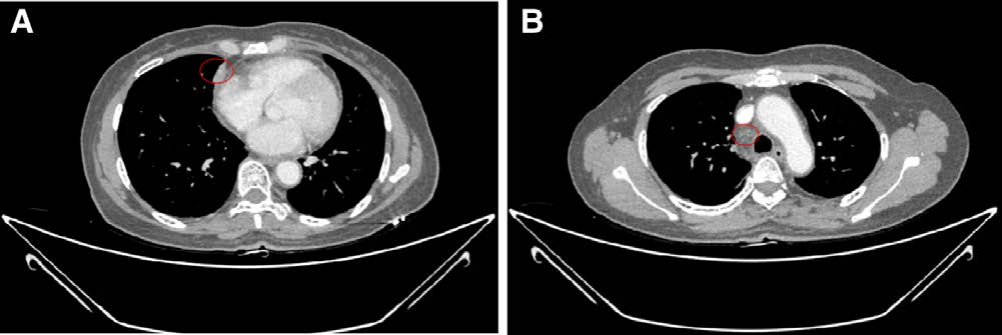
Follow-up results on March 23, 2023. (A) Stable primary lesion; (B) no enlargement of the mediastinal lymph nodes.

Follow-up chest CT indicated that the primary lesion and mediastinal lymph nodes remained stable (Fig. 10A and B). The dosing interval for camrelizumab was gradually extended, and the treatment regimen was adjusted to camrelizumab 0.2 g every 8 weeks (q8w), combined with anlotinib 12 mg daily from Day 1 to Day 14 (d1–d14).

#### 2.4.10. June 21, 2023

**Figure 11.**
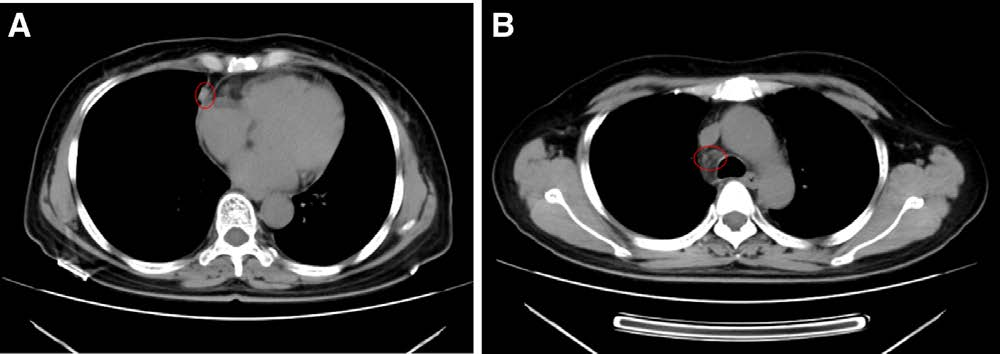
Follow-up results on June 21, 2023. (A) Stable primary lesion; (B) no enlargement of the mediastinal lymph nodes.

A follow-up chest CT indicated stable disease, and no metastases were observed in abdominal CT (Fig. 11A and B).

#### 2.4.11. November 2, 2023

**Figure 12.**
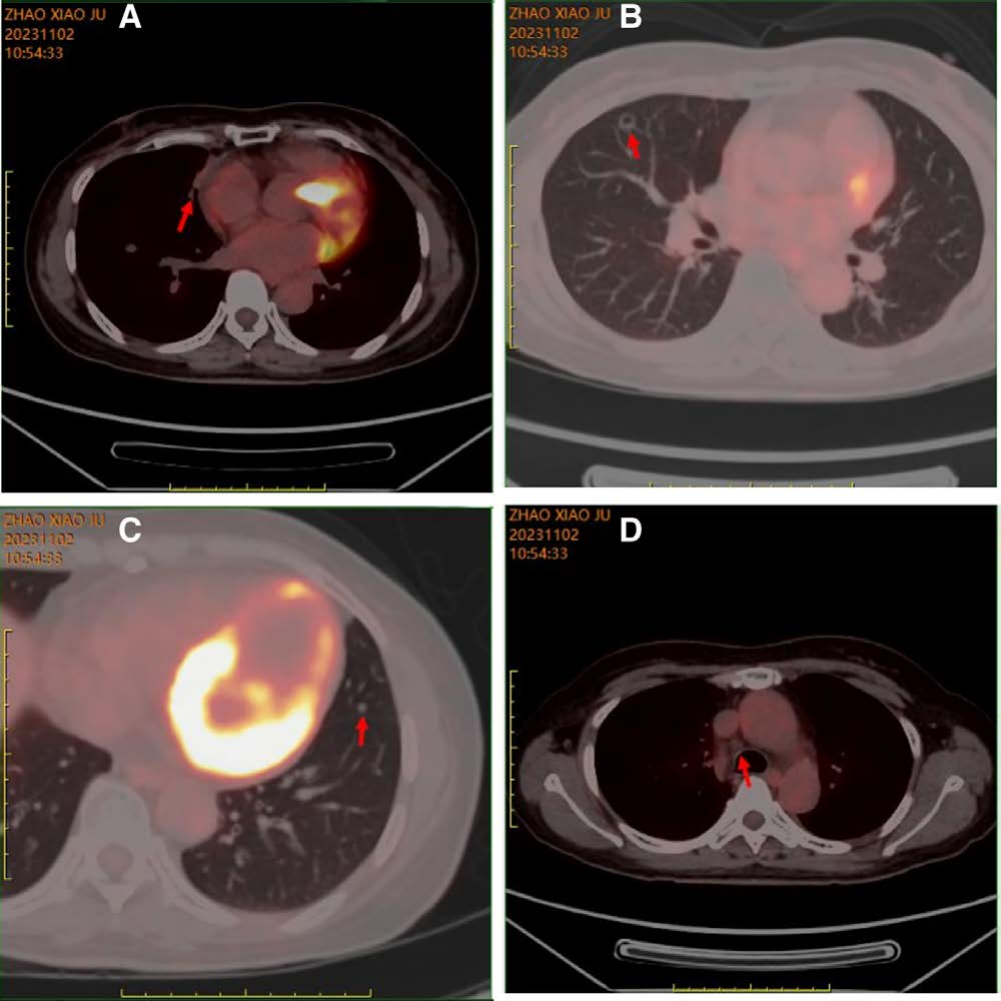
PET-CT Results on November 2, 2023. (A) No increase in the size of the primary lesion; (B) Stable treated metastatic lesion; (C) Stable treated metastatic lesion; (D) No enlargement of the mediastinal lymph nodes. CT = computed tomography, PET = positron emission tomography.

A comprehensive whole-body positron emission tomography-CT scan revealed: The SCC in the right middle lung, after comprehensive treatment, had reduced to a size of approximately 1.7 cm × 0.8 cm (coronal view) with an SUVmax of 1.4. A small area of increased density was observed in the right middle lung, with mild metabolic activity, suggesting that the tumor was under clear suppression. Multiple nodules in the right lung and left lung lower lobe (maximum size approximately 0.9 cm × 0.9 cm) showed no metabolic increase, indicating that they were stable metastatic lesions after treatment. A small lymph node in the mediastinum (4R) showed no metabolic increase (size approximately 0.7 cm × 0.6 cm), suggesting that the metastatic lesion in the lymph node was under clear suppression after treatment (Fig. 12A–D).

### 2.5. Surveillance

#### 2.5.1. On November 7, 2024, the most recent follow-up and treatment

**Figure 13.**
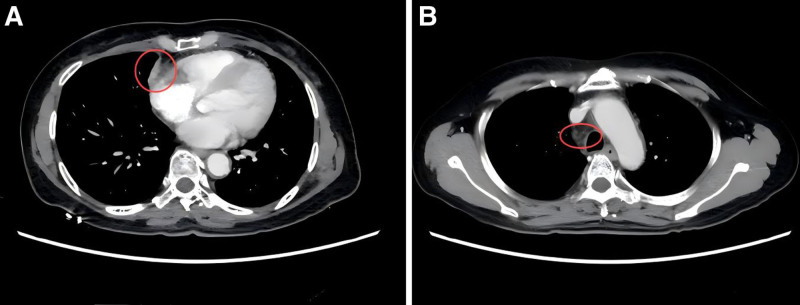
Chest CT follow-up on November 7, 2024. (A) Right lung lesion remains stable; (B) mediastinal metastatic lymph nodes remain largely unchanged in size. CT = computed tomography.

The patient continues to receive stable treatment with the combination of Camrelizumab (0.2 g every 8 weeks) and Anlotinib (12mg daily from day 1 to day 14). Chest contrast-enhanced CT revealed that the lung tumor (approximately 15 mm × 4 mm) and mediastinal lymph nodes were generally unchanged (Fig. 13A and B), with the right middle lung target lesion showing a more than 30% reduction in size. No new tumor progression was observed. Brain MRI showed no significant abnormalities, and abdominal CT did not reveal any metastatic lesions. The treatment outcome was evaluated as partial response. The patient’s current OS is approximately 59 months, and the total PFS (Total PFS) for second-line treatment is approximately 51 months. As of the date of the last follow-up, the treatment remains effective.

### 2.6. Adverse reactions

Throughout the extended course of combination therapy with camrelizumab and anlotinib, initiated January 20, 2021, the patient’s tolerance was generally favorable, although predictable treatment-related adverse events (TRAEs) emerged and were meticulously managed. Approximately 6.5 months posttreatment initiation (August 9, 2021), the patient developed grade 2 hypothyroidism, characterized by laboratory findings of decreased free T3 (2.0 pmol/L; reference range: 2.1–5.4 pmol/L) and free T4 (8.3 pmol/L; reference range: 9–25 pmol/L) with a concomitant elevation of thyroid-stimulating hormone (9.8 mIU/L; reference range: 0.3–4.8 mIU/L).^[25]^ This endocrinopathy is a well-documented and “very common” immune-related adverse event consistent with camrelizumab’s established immunotoxicity profile. It was promptly addressed with daily oral levothyroxine sodium supplementation (100 mg), leading to the normalization of thyroid parameters within 4 weeks (TSH: 3.2 mIU/L; free T4: 14.5 pmol/L) and sustained euthyroidism under regular monitoring. Subsequently, at 8.5 months post-initiation (October 11, 2021), Grade 2 hypertension manifested (blood pressure: 162/105 mm Hg; grade 2 range: systolic blood pressure 160–179 or diastolic blood pressure 100–109 mm Hg).^[26]^ This finding aligns with the established safety profile of anlotinib, for which hypertension is recognized as a frequent adverse effect. Antihypertensive intervention with daily oral amlodipine (5 mg) effectively normalized blood pressure values within 1 week, with stability maintained via a home monitoring protocol. Crucially, both hypothyroidism and hypertension are recognized, common side effects associated with camrelizumab and anlotinib, respectively. Their occurrence in this case was therefore anticipated and managed using standard clinical practices. Importantly, neither of these events (grade 2 hypothyroidism and grade 1 hypertension) necessitated dose modification, interruption, or cessation of the camrelizumab plus anlotinib regimen, nor did they significantly compromise the patient’s functional status (maintained at ECOG 1) or overall quality of daily life. The absence of other clinically significant toxicities, such as pneumonitis, colitis, or hepatitis, further underscores the manageable safety profile observed in this patient, highlighting that vigilant monitoring and appropriate supportive care can facilitate treatment continuity with this combination therapy.

### 2.7. Ethical statement

This case report was approved by the Ethics Committee of Maoming People’s Hospital (PJ2024MI-K157-01). The patient provided informed consent for the publication of this report, and all personal information has been anonymized to protect patient confidentiality.

## 3. Literature review

Camrelizumab is a humanized monoclonal antibody and an immune checkpoint inhibitor that primarily exerts its effect by targeting programmed cell death protein 1 (PD-1). Compared to other PD-1 inhibitors such as nivolumab and pembrolizumab, camrelizumab differs in its molecular structure, particularly with modifications to its Fc region. These modifications enhance antibody-dependent cell-mediated cytotoxicity (ADCC), allowing camrelizumab to more effectively activate the immune system and thereby demonstrate improved therapeutic efficacy.^[27]^ The production process of camrelizumab utilizes an advanced expression system, ensuring its stability and purity during large-scale production while maintaining high biological activity over prolonged storage.^[28]^ Consequently, camrelizumab offers a stronger immune response and better clinical outcomes when used to treat various cancers, including NSCLC.

Currently, clinical case reports on the combination of camrelizumab and anlotinib for NSCLC treatment remain limited. A search of the PubMed database yielded 2 relevant case reports^[29,30]^ (Table 3), though several clinical studies have provided preliminary data supporting the potential of this combination strategy in NSCLC treatment.

**Table 3:** Summary of case reports on the combination of camrelizumab and anlotinib in the treatment of NSCLC.

Reference	Age/gender	Histology	Line of treatment	Treatment regimen	Efficacy
Qi et al^[29]^	74 yr/male	Adenocarcinoma	First-line	Camrelizumab 200 mg IV on day 1 + Anlotinib 12 mg PO QD days 1 to 14/Q3W	PR, PFS > 19 mo
Lei et al^[30]^	55 yr/male	Pulmonary lymphoepithelioma	Third-line	Camrelizumab 200 mg IV on day 1 + Anlotinib 12 mg PO QD days 1 to 14/Q3W	PR, PFS > 26 mo

IV = intravenous (administered into a vein), NSCLC = non-small-cell lung cancer, PFS = progression-free survival, PO = per Os (latin for “by mouth,” meaning oral administration), PR = partial response, Q3W = every 3 wk (indicating the frequency of administration, every 3 wk), QD = quaque die (latin for “once a day,” meaning daily).

A retrospective study analyzed 139 advanced NSCLC patients who received at least 1 dose of ICIs combined with anlotinib (IA group) or anlotinib alone (AA group). The results showed that the median PFS (mPFS) was significantly longer in the IA group compared to the AA group (IA: 5.8 months vs AA: 4.2 months, *P* = .022), with a hazard ratio (HR) of 0.68 (95% CI: 0.68–0.97). Particularly in patients with brain metastases, the IA group demonstrated greater efficacy (mPFS: 6.0 months vs 3.8 months, *P* = .034), with an HR of 0.49 (95% CI: 0.23–1.05). These results suggest that the combination of ICIs and anlotinib significantly enhances efficacy, especially in controlling intracranial lesions.^[31]^ However, some studies have indicated that no statistically significant difference in mPFS was observed when combining immunotherapy with anlotinib compared to anlotinib alone. A retrospective study evaluated the efficacy and safety of anlotinib combined with immunotherapy in 80 advanced NSCLC patients (median age 62 years). The overall median PFS was 4.3 months (95% CI: 2.7–5.9 months). For patients receiving the combination therapy, the median PFS was slightly longer than that of those receiving anlotinib alone (4.2 months vs 3.1 months), although the difference did not reach statistical significance. Notably, in patients without EGFR mutations, no prior EGFR-targeted therapy, and no brain metastasis, the median PFS was significantly prolonged. These findings suggest that the combination of anlotinib and immunotherapy offers some efficacy for advanced NSCLC patients and is well tolerated.^[32]^

The CameL-Sq trial, a pivotal phase III study, evaluated the efficacy and safety of camrelizumab combined with carboplatin and paclitaxel as first-line treatment for advanced squamous NSCLC. The results demonstrated that the camrelizumab combination group significantly outperformed the placebo group in both median PFS and median OS. Specifically, the camrelizumab combination group had a median OS of 27.4 months, a 11.9-month improvement over the chemotherapy-only group (*P* < .0001); the 4-year OS rate was 33.9%, with an HR of 0.56 (95% CI: 0.45–0.72), showing statistical significance (*P* < .0001). The 4-year PFS rate was 20.5%, which was significantly longer than that of the chemotherapy-only group, with a 5.6-month improvement and a 68% reduction in the risk of progression. Furthermore, biomarker analysis revealed that the clearance of ctDNA after 2 cycles of treatment was closely associated with prolonged PFS and OS. These results provide robust clinical evidence supporting the application of camrelizumab combined with chemotherapy in advanced squamous NSCLC patients.^[33]^

Additionally, the SHR-1210-201 trial, a phase II study, evaluated the efficacy and safety of camrelizumab in advanced or metastatic NSCLC patients with varying levels of PD-L1 expression. A total of 146 patients were enrolled, all receiving camrelizumab (200 mg, intravenous infusion, every 2 weeks). The study found an overall ORR of 17.8% (95% CI: 12.0%–25.0%), with response rates improving as PD-L1 TPS increased. Specifically, the ORR in patients with PD-L1 TPS < 1%, 1% to 25%, 25% to 50%, and ≥ 50% was 12.2%, 19.4%, 36.4%, and 23.3%, respectively. The median PFS was 3.2 months (95% CI: 2.0–3.4), and the median OS was 14.8 months (95% CI: 10.2–18.7). Additionally, the 5-year OS rate was 19.3%. Camrelizumab showed a favorable safety profile, with TRAEs occurring in 87.7% of patients, and 21.2% of patients experiencing grade 3 or higher TRAEs. This study further supports the effectiveness of camrelizumab in second-line treatment for advanced NSCLC, particularly in patients with PD-L1 positivity.^[34]^ A retrospective cohort study evaluating this combination as second-line therapy for extensive-stage small-cell lung cancer (ES-SCLC) demonstrated a pronounced and statistically significant improvement in PFS compared to chemotherapy (median PFS: 7.0 vs 3.0 months; HR: 0.34, 95% CI: 0.15–0.77; *P* < .001). While this robust efficacy signal did not translate into a statistically significant OS benefit in this cohort (median OS: 16.3 vs 17.3 months; *P* = .82), the combination yielded numerically higher ORRs (ORR: 52.9% vs 23.5%) and disease control rates (82.4% vs 58.8%), alongside a favorable safety profile marked by fewer grade 3 or higher adverse events compared to chemotherapy (17.6% vs 29.4%), suggesting substantial antitumor activity and manageability in this challenging setting.^[35]^ Further supporting its utility in heavily pretreated patients, another study focused on third-line advanced NSCLC found that adding camrelizumab to anlotinib significantly improved the “total effective rate” (*P* < .05), reduced serum tumor marker levels (*P* < .001), mitigated cancer-related fatigue (CFS score, *P* < .001), and enhanced PS (KPS score, *P* < .001) compared to anlotinib monotherapy, remarkably achieving this with a lower overall incidence of adverse reactions (*P* < .05).^[36]^ Expanding the scope beyond pulmonary malignancies, the combination has also shown promise in retroperitoneal soft tissue sarcomas (RSTs), a distinct cohort where conventional therapies offer limited benefit. A single-center retrospective analysis of 57 RST patients treated with camrelizumab plus anlotinib revealed encouraging activity, with an overall ORR of 26.3% (including 3.5% complete responses), a disease control rates of 80.7%, and a median PFS of 9.1 months. Notably, this study highlighted differential responses based on histology, with significantly better ORR (52.6% vs 13.2%; *P* = .0031) and median PFS (11.1 vs 6.3 months; *P* = .0256) observed in non-L-sarcomas compared to L-sarcomas. The safety profile in this sarcoma cohort was consistent with known toxicities, with hypertension (24.6%), hypothyroidism (19.3%), and palmar-plantar erythrodysesthesia (12.3%) being the most common grade 3 to 4 TRAEs occurring in 22.8% of patients.^[37]^

In conclusion, the research on camrelizumab combined with anlotinib for treating NSCLC has progressively shown promising results. Existing data support the potential of this combination therapy in improving patient survival and delaying disease progression, while also demonstrating a favorable safety profile. Future clinical trials and real-world data are necessary further to validate its broader application in various clinical settings.

## 4. Discussion

Immune checkpoint inhibitors have become the standard first-line treatment for advanced NSCLC with negative driver mutations, significantly improving patient survival. Particularly in patients with high PD-L1 expression, the 5-year survival rate of immunotherapy can reach 40%.^[38]^ However, despite the significant efficacy demonstrated by immunotherapy in the initial treatment phase, second-line treatment options after immunotherapy resistance and tumor progression remain limited. The current guidelines from the NCCN and the Chinese Society of Clinical Oncology both recommend chemotherapy, such as docetaxel, as the standard second-line treatment option. The TAX320 trial demonstrated that docetaxel achieved an ORR of only 10.8% in pretreated advanced NSCLC patients.^[39]^ Additionally, the TAX317 trial indicated that docetaxel as second-line treatment extended the median OS by only 2.4 months compared to best supportive care.^[40]^ These results highlight the limited efficacy of traditional second-line monotherapy, underscoring the urgent need for more effective treatment strategies following immune therapy resistance.

In recent years, the combination of anti-angiogenic drugs and ICIs has gained widespread attention. Angiogenesis plays a critical role in tumor initiation and progression. Overactivation of the vascular endothelial growth factor (VEGF) signaling pathway can inhibit the expression of intercellular adhesion molecule-1 (ICAM-1) and vascular cell adhesion molecule-1 (VCAM-1), thereby suppressing the transport of immune cells to the tumor site and promoting immune evasion by the tumor.^[41]^ Anti-angiogenic therapy, which targets multiple pathways and multiple targets of VEGF, has been shown to effectively improve the tumor microenvironment, enhance immune cell infiltration into tumor tissues, and strengthen the immune system’s antitumor capacity. Animal model studies have demonstrated that inhibition of VEGF and its related pathways normalizes tumor vasculature, reprogramming the tumor microenvironment from an immune-suppressive state to an immune-activated state.^[42]^ Moreover, anti-angiogenic therapy can increase the formation of high endothelial venules, thereby enhancing tumor sensitivity to immunotherapy.^[43]^ Therefore, the combination of anti-angiogenic drugs with ICIs may enhance the effectiveness of immunotherapy by improving the tumor microenvironment, making it a promising therapeutic strategy.

The LUNG-MAP S1800A trial studied advanced or relapsed NSCLC patients who had previously received immunotherapy. The trial compared the efficacy of pembrolizumab plus lenvatinib with standard chemotherapy regimens (such as docetaxel, gemcitabine, etc). The results showed that the combination of pembrolizumab and lenvatinib significantly improved OS (median OS 14.5 months vs 11.6 months in the standard treatment group, *P* < .0001).^[44]^ This further validates the potential of combining ICIs with anti-angiogenic drugs in NSCLC.

In the case presented in this report, the patient, after progressing on first-line treatment with pembrolizumab plus chemotherapy, received platinum-based doublet chemotherapy combined with radiotherapy. Following subsequent progression, the patient achieved over 4 years of long-term survival with the combination of camrelizumab and anlotinib. This outcome may be attributed to the pharmacological mechanisms of camrelizumab and anlotinib. Camrelizumab, a humanized monoclonal antibody targeting the programmed cell death protein 1 (PD-1), plays a crucial role in enhancing T-cell-mediated antitumor immune responses. By blocking the interaction between PD-1 and its ligands PD-L1 and PD-L2, often expressed on tumor cells and other immune-regulatory cells within the tumor microenvironment (TME), camrelizumab effectively reverses the immune suppression camrelizumab effectively reverses immune suppression and reactivates exhausted T lymphocytes, thereby promoting a robust antitumor immune response^[29,45]^. This blockade leads to the activation of cytotoxic T lymphocytes, which are essential for mounting an effective immune response against tumor cells.^[46,47]^ However, the efficacy of single-agent ICIs like camrelizumab can be constrained by the complex TME, particularly in “cold” tumors lacking immune infiltrate or where multiple immunosuppressive mechanisms (e.g., hypoxia, aberrant angiogenesis, inhibitory cell populations like Tregs and MDSCs) establish barriers to primary or acquired resistance.^[45]^ Indeed, camrelizumab-based regimens have demonstrated the capacity to reinvigorate immune surveillance and enhance cytotoxic T-cell activity across various malignancies, including advanced lung cancer, even where monotherapy proved insufficient, highlighting the critical need for strategies that overcome these TME-associated limitations.^[30,35]^. On the other hand, anlotinib, a multi-target small-molecule tyrosine kinase inhibitor with potent anti-angiogenic properties, complements the immune-enhancing effects of camrelizumab by profoundly modulating the TME. Anlotinib inhibits various angiogenic signaling pathways, including those mediated by VEGF and fibroblast growth factor (FGF), which are pivotal in tumor growth and metastasis.^[48]^ Crucially, the inhibition of VEGFR-mediated signaling not only deprives tumors of essential blood supply via antiangiogenesis but also promotes the “normalization” of the tortuous and leaky tumor vasculature.^[36]^ This vascular normalization improves tissue perfusion, alleviates intratumoral hypoxia – a potent immunosuppressive stimulus – and potentially reduces the recruitment of inhibitory immune cells. Furthermore, normalized vessels exhibit improved barrier function and may upregulate adhesion molecules, thereby enhancing the infiltration of activated T cells, such as those unleashed by camrelizumab, into the tumor core.^[49]^ Anlotinib might also counteract resistance by inhibiting alternative angiogenic pathways contributing to the immunosuppressive milieu.^[50]^ The normalization of tumor blood vessels not only enhances immune cell infiltration but also facilitates the effective delivery of therapeutic agents, thereby improving the overall efficacy of cancer treatments.^[51]^ The combination therapy not only enhances the immune system’s ability to recognize and kill tumor cells but also improves tumor sensitivity to immunotherapy by restoring angiogenesis and modulating the tumor microenvironment, thus extending the patient’s survival. Critically, the integration of camrelizumab and anlotinib leverages distinct yet complementary mechanisms to potentially achieve synergistic effects and overcome immunotherapy resistance. This strategy rests on several pillars: First, while camrelizumab “releases the brakes” on T cells by blocking PD-1, anlotinib actively remodels the TME – normalizing vasculature, reducing hypoxia, and lowering interstitial fluid pressure – creating a more permissive environment for these activated T cells to infiltrate and exert their cytotoxic functions.^[37]^ Second, by targeting multiple pathways (VEGFR, FGFR, PDGFR), anlotinib provides a broader countermeasure against diverse immunosuppressive signals mediated by these pathways, potentially mitigating resistance mechanisms that extend beyond the PD-1/PD-L1 axis. For instance, by limiting aberrant angiogenesis, anlotinib might indirectly reduce the secretion of immunosuppressive cytokines like IL-6, implicated in sustaining resistance to PD-1 blockade.^[50]^ Third, accumulating clinical evidence from case reports and retrospective studies indicates enhanced efficacy and prolonged PFS with the combination therapy compared to monotherapies, suggesting this dual-targeting approach effectively counters a spectrum of resistance mechanisms.^[52]^ Notably, beyond enhancing antitumor immunity, the combination might offer improved tolerability; the anti-angiogenic action of anlotinib may alleviate certain camrelizumab-specific adverse events, such as reactive cutaneous capillary endothelial proliferation, thereby potentially enhancing patient compliance while maintaining therapeutic efficacy.^[53]^

From a pathological perspective, the tumor in this patient was SCC, a subtype of NSCLC often associated with high PD-L1 expression. PD-L1-positive tumor cells can inhibit T-cell activity by binding to PD-1. Camrelizumab restores T-cell function by blocking the PD-1/PD-L1 interaction, thereby enhancing the immune response. The patient’s genetic testing results revealed negative driver mutations, further supporting the rationale for using ICIs in combination with anti-angiogenic therapy. The combined treatment strategy not only takes advantage of the immune checkpoint inhibitor’s ability to restore immune function but also utilizes the anti-angiogenic drug to improve the tumor microenvironment, providing better penetration and activation of immune cells, thus enhancing the therapeutic effect.

## 5. Conclusion

In summary, the combination of camrelizumab and anlotinib has demonstrated promising efficacy in advanced SCC of the lung, particularly in patients with immune therapy resistance. This approach shows potential for broader application, especially in the context of overcoming immune treatment resistance. The combined use of immunotherapy and anti-angiogenic treatment may help improve the tumor microenvironment from multiple aspects, enhancing the efficacy of immunotherapy and providing a potential new treatment strategy for advanced NSCLC. However, it is important to note that this is based on a single case report, and further clinical studies and real-world data are necessary to validate the long-term efficacy and safety of this treatment regimen.

## Acknowledgments

We would like to thank Zhibin Xu from the The First Affiliated Hospital of Guangzhou Medical University for assisting with the preparation and English revision of this manuscript.

## Author contributions

**Conceptualization:** Chunning Zhang.

**Data curation:** Manjie Li, Chunning Zhang, Yongquan Deng.

**Methodology:** Manjie Li, Chunning Zhang, Junfen Cheng.

**Project administration:** Caiping Ke.

**Software:** Qiwen Huang.

**Supervision:** Junfen Cheng.

**Validation:** Qiwen Huang.

**Visualization:** Caiping Ke, Qiwen Huang.

**Writing – original draft:** Caiping Ke, Junfen Cheng.

**Writing – review & editing:** Caiping Ke, Junfen Cheng.

## Correction

This article was originally published with affiliations “a & b” ^a^ (Department of Oncology, Unit 1, Maoming People’ s Hospital, Maoming, Guangdong Province, China) and ^b^ (The First School of Clinical Medicine, Guangdong Medical University, Zhanjiang, Guangdong Province, China) have been now updated online as follows ^a^ (The First School of Clinical Medicine, Guangdong Medical University, Zhanjiang, Guangdong Province, China) and ^b^ (Department of Oncology, Unit 1, Maoming People’ s Hospital, Maoming, Guangdong Province, China). In this process, the affiliation links of Authors Dr. Zhang and Dr. Deng have also been changed from “a” to “b” to match with the existing order of affiliations.

## Supplementary Material


