# Supplementary figures and images for: Immune checkpoint inhibitors combined with targeted therapy for long-term survival in advanced pulmonary squamous cell carcinoma after first-line failure: A case report and literature review
Source: Medicine (Baltimore). 2025 Jun 20;104(25):e42724. doi: 10.1097/MD.0000000000042724 (PMC12187345; doi:10.1097/MD.0000000000042724)

**Supplementary Figure 1 Clinical Pathway Timeline Flowchart**


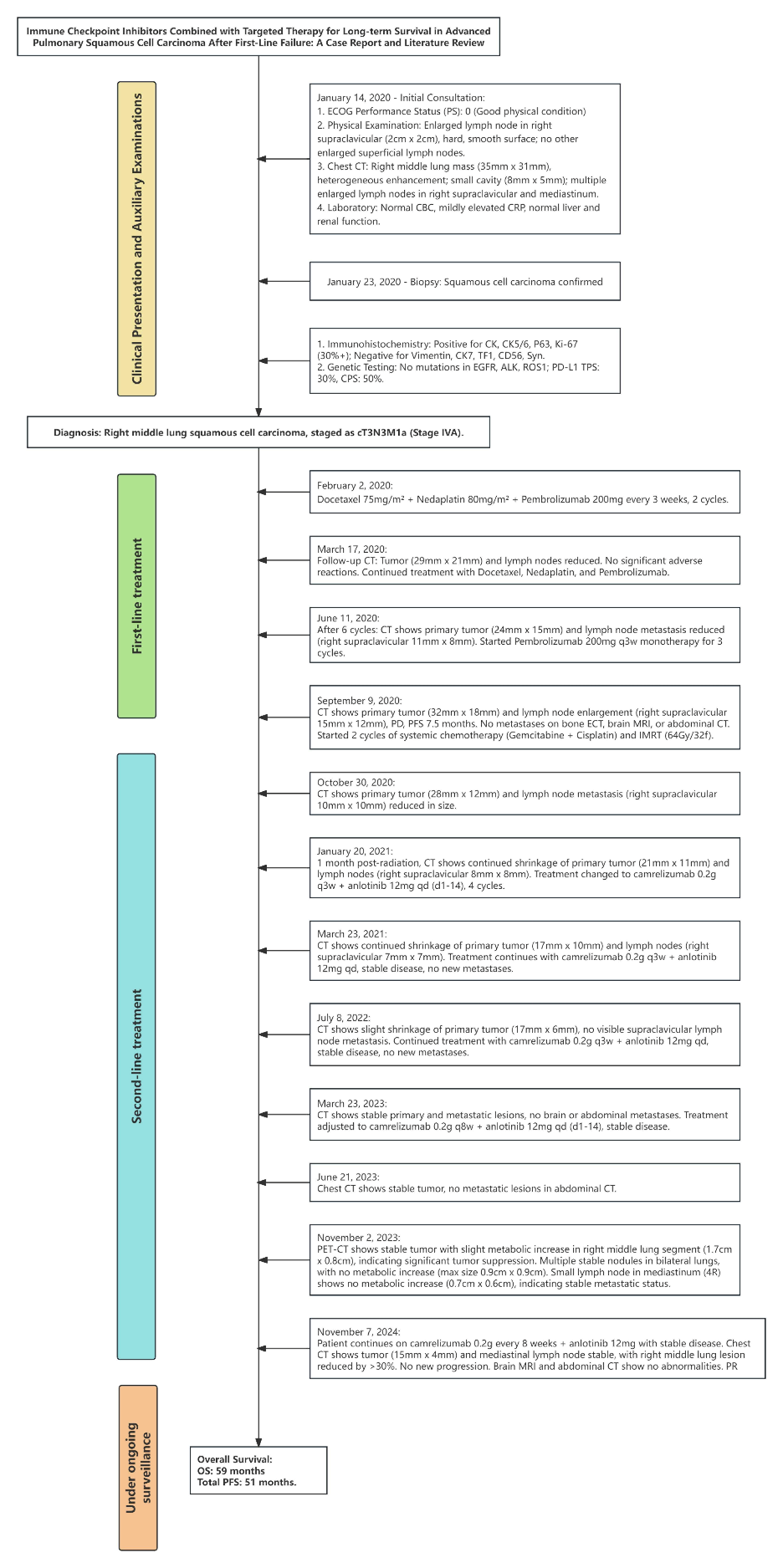

Supplement: Supplementary file 1 [file medi-104-e42724-s001.docx]
